# Implementation of a successful eradication protocol for *Burkholderia Cepacia* complex in cystic fibrosis patients

**DOI:** 10.1186/s12890-018-0594-8

**Published:** 2018-02-14

**Authors:** Bryan A. Garcia, Jacque L. Carden, Dana L. Goodwin, Tim A. Smith, Amit Gaggar, Kevin Leon, Veena B. Antony, Steven M. Rowe, George M. Solomon

**Affiliations:** 1 Department of Medicine, Division of Allergy, Pulmonary and Critical Care Medicine, 1900 University Blvd, THT 422, Birmingham, AL 35294 USA; 2 Gregory Fleming James Cystic Fibrosis Research Center, Birmingham, AL USA

**Keywords:** *Burkholderia cepacia*, *Burkholderia Cepacia complex (BCC)*, Cystic fibrosis, Infection eradication

## Abstract

**Background:**

Infection with *Burkholderia cepacia complex* (*Bcc*) results in a heterogeneous clinical course ranging from asymptomatic colonization of the airways to fulminant respiratory failure in patients with cystic fibrosis (CF). Early eradication of *Pseudomonas aeruginosa* improves clinical outcomes. The efficacy and clinical outcomes following implementation of an eradication protocol for *Bcc* are less well understood.

**Methods:**

We developed and implemented a single center *Bcc* eradication protocol that included an intensive combination of intravenous, inhaled, and oral antibiotic therapies based on in vitro sensitivities. We conducted a retrospective cohort analysis of clinical outcomes compared to patients with chronic *Bcc* infection.

**Results:**

Six patients were identified as having a newly acquired *Bcc* colonization and were placed on the eradication protocol. Sequential sputum samples after completion of the protocol demonstrated sustained clearance of *Bcc* in all patients. Lung function and nutritional status remained stable in the year following eradication.

**Conclusion:**

Clearance of *Bcc* from sputum cultures using a standardized protocol was successful at one year and was associated with clinical stability.

## Background

Cystic Fibrosis (CF) is characterized by development of viscous airway mucus which provides a fertile media for opportunistic respiratory pathogens [1]. Chronic respiratory infection with recurrent exacerbations and inflammation results in progressive bronchiectasis and ultimately respiratory failure [2, 3]. Aggressive management of chronic respiratory infections is crucial to prevent lung function decline. *Pseudomonas aeruginosa* is a common respiratory pathogen in the CF airway and chronic colonization is associated with impaired lung function for these patients [4, 5]. Early eradication upon initial positive sputum culture with *P. aeruginosa* has been shown to stabilize lung function and improve clinical outcomes with successful eradication ranging from 62 to 93% although the long-term clinical benefit may be uncertain [6–8].

Though less prevalent than *P. aeruginosa*, CF patients may acquire other respiratory pathogens including species of the *Burkholderia cepacia* complex *(Bcc)* [9]. *Bcc* represents a group of genetically related bacteria associated with a heterogeneous clinical course ranging from asymptomatic colonization to fulminant respiratory failure [10, 11]. Chronic colonization with *Bcc* is associated with antibiotic resistance, increased risk of respiratory failure, and worsened mortality [10, 12]. Although *Bcc* eradication has been previously described, implementation of a protocolized approach toward *Bcc* eradication has not been fully studied [13, 14].

## Methods

### Patient identification and data collection-

We obtained institutional review board approval to conduct a retrospective chart review of CF patients infected with *Bcc* at our center. Beginning January 2012 through June 2015, patients were placed on the eradication protocol if they were found to have a sputum culture positive for *Bcc,* which had not been isolated from sputum during the two prior years. Patients who were known to have chronic airway colonization by *Bcc* were treated with guideline-based standard of care [15]. Lung function (forced expiratory volume in one second, FEV_1_% predicted), nutritional status (Body mass index, BMI), and serial sputum cultures were obtained during clinic appointments that generally occurred at 3-month intervals, per CF guidelines. The genomovar of *Bcc* strains isolated was confirmed at the *B. cepacia* Research Laboratory and Repository at the University of Michigan. Successful eradication of *Bcc* was defined as clearance of *Bcc* following the induction stage of therapy with maintenance of at least three additional negative respiratory cultures at 3-month intervals during the 12-month follow-up.

### Treatment protocol

Patients included in the eradication group following sputum culture isolation of newly acquired *Bcc* were placed on the eradication protocol (Table 1). Antibiotic selection was based on identifying an aggressive combination of inhaled and intravenous therapies with broad coverage to obtain maximal in-vivo treatment synergy as multiple patient strains demonstrated pan-resistance based on in-vitro antibiotic sensitivity testing. Tobramycin was included for empiric coverage of *P. aeruginosa* given the high rate of co-infection in this population. The rationale for antibiotic selection is included in Table 1.

**Table 1:** *Burkholderia* Eradication Protocol

	Medication	Dosing	Frequency	Route	Bacteria Targeted
Induction Period (21 Days):	Tobramycin	6 mg/kg (per kinetics)	Daily	IV	*PsA, Bcc*
	Ceftazidime	2 g	Every 8 h	IV	*PsA, Bcc*
	Trimethoprim/Sulfamethoxazole	800/160 mg	Twice Daily	Oral	*Bcc*
	Tobramycin inhaled^a^	300 mg	Twice Daily	Nebulized	*PsA, Bcc*
	Azithromycin	250 mg	Daily	Oral	Anti-inflammatory
Consolidation Period (2 months):	Trimethoprim/Sulfamethoxazole	800/160 mg	Twice daily	Oral	*Bcc*
	Tobramycin inhaled^a^	300 mg	Twice daily	Nebulized	*PsA, Bcc*
	Azithromycin	250 mg	Daily	Oral	Anti-inflammatory

^a^Alternative- TIP (tobramycin dry powder for inhalation, 4 caps (28 mg/cap)) every 12 h

### Statistical analysis

Statistical analysis was performed using GraphPad Prism version 6.0c (GraphPad Software, Inc., San Diego, CA). Continuous variables were described using means with standard deviations, or medians with ranges as sample sizes were small, and were analyzed using Mann-Whitney *t*-test or Two-way ANOVA testing as data were non-parametric. Change in lung function and nutritional status was compared between groups using Two-way ANOVA; Fisher’s post hoc test was used when ANOVA was significant. All statistical tests were two-sided and *P*-values < 0.05 were considered statistically significant.

## Results

### Patient cohort

From January 2012 to June 2015, 14 adult patients were found to have *Bcc* isolated in sputum cultures. Seven of these patients were found to have a newly acquired *Bcc* colonization (no isolation of *Bcc* from sputum in the two preceding years), of which six were placed on eradication protocol. The remaining patient was not included in analysis due to a new acquisition of *P. aeruginosa* during the maintenance phase of the *Bcc* eradication, requiring transition to a *PsA* eradication protocol. Seven additional patients were defined as having chronic colonization with *Bcc* based on prior positive cultures for *Bcc*; these patients were treated based on guidelines for pulmonary exacerbation and were considered as contemporaneous controls. Of these seven control patients, two patients were not included due to inadequate clinic follow up. A third patient with *Bcc* colonization was not included due to identification of only one CF causing mutation despite genetic sequencing. These patients were excluded from further analysis. This control group thus consists of patients with chronic *Bcc* infection and act as case controls for patients with acute infection.

### *Outcome following* bcc *Eradication*

Demographic data for the two groups including age, gender, *CFTR* genotype, baseline lung function, nutritional status, pancreatic functional status, and microbiology culture results at baseline were collected via chart review (Table 2). All patients in both groups were maintained on thrice weekly oral azithromycin therapy for anti-inflammatory effect. In addition, all patients placed on *Bcc* eradication were previously colonized with *PsA* and were maintained on every-other-month inhaled tobramycin. Three of the four control patients were also previously maintained on inhaled tobramycin for chronic *PsA* infection.

**Table 2:** Baseline characteristics

Group	Gender	Age	Genotype (n)	Pancreatic Insufficient (%)	CFRD (%)	Bcc Species Isolated (*n*)	Additional Pathogens (*n*)	FEV_1_% Predicted	BMI
Eradication (*n* = 6)	50% F	23.3 (20–27)	dF508/dF508 [6]	(100%)	83%	B. multivorans [4] B. gladioli [2] *B. cepacia* [1]	PsA [6] MRSA [3] Steno [2] Aspergillus [2]	65.5 (30–96%)	20.2 (15.6–26.9)
Usual Care (*n* = 4)	50% F	28.2 (21–47)	dF508/dF508 [1] 405 3AtoC/V317A Q98X [1] dF508/Q98X [1] dF508/G542X [1]	(75%)	75%	B. multivorans [1] B. cepacia [2] B. arboris [1]	PsA [3] MRSA [4]	55.7 (31–98%)	20.3 (18.9–22)

Abbreviations: *F*=Female, *CFRD*- CF Related Diabetes, *MRSA*- Methicillin Resistant *Staphylococcus aureus*, *PsA*- Pseudomonas aeruginosa, Steno- *Stenotrophomonas maltophilia*

**Fig. 1:**
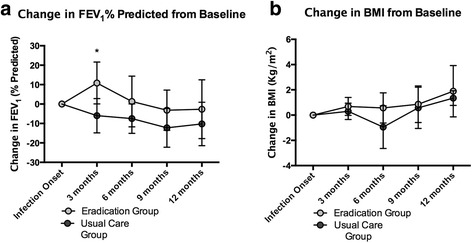
**a**. Change in mean lung function (FEV_1_% predicted) from baseline over time during the course of the cohort study for patients in the eradication group compared to patients treated with standard of care. *p = 0.01. **b**. Change in mean BMI from baseline for the same period

At baseline there was no significant difference between the group means of the eradication and control groups for FEV_1_% predicted (65.5 ± 24.5% vs. 59.7 ± 28.2%, respectively, *p* = 0.67) or BMI (20.2 ± 3.8 vs. 20.3 ± 1.2, respectively, *p* = 0.94). Among patients in the eradication group, 100% of the patients remained free of *Bcc* at the end of the study compared to 25% in the control group. During the one-year follow up period the eradication group experienced a mean number of exacerbation of 1.66 ± 0.42 versus 3.25 ± 1.0 by the control group (*p* = 0.166). The mean time to first exacerbation was 230 days (± 24.55) in the eradication group versus 88 ± 27.6 in the control group (*p* = 0.009). The change from baseline between the eradication and control groups was compared and at three months the groups had a significant difference in change of FEV_1_% predicted (10.8 ± 10.7% vs. -6.0 ± 8.8%, *p* = 0.01) (Fig. 1). No significant difference in change of FEV_1_% predicted was identified between the eradication and control groups at 6 (1.3 ± 13.0% vs. -7.5 ± 7.5%) or 12 months (− 2.6 ± 15.1% vs. -10.2 ± 11.1%). With regards to nutritional status, no significant difference was identified in change in BMI from baseline between the eradication and control groups at three (0.68 ± 0.71 vs. 0.3 ± 0.64 kg/m^2^), six (0.56 ± 1.1 vs. -0.95 ± 1.6 kg/m^2^), or 12 months (1.8 ± 2.0 vs. 1.3 ± 0.5 kg/m^2^).

**Fig. 2:**
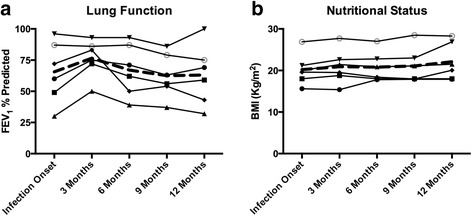
**a**. Trends in lung function (FEV_1_% predicted) for each eradication group subject over time (*n* = 6 patients). **b**. Trends in nutritional status (BMI) for each eradication group subject over time (*n* = 6 patients). Dashed lines represent the trend of the group mean over time in each graph

We also report absolute FEV_1_% and BMI in the eradication group by individual. Among these patients, no significant difference was seen in mean (± standard deviation) FEV_1_% predicted at three (76.5 ± 15.0), six (67 ± 20.9), and 12 months (63 ± 24.1) following eradication compared to either baseline (65.6 ± 24.4) or the average of the year prior to *Bcc* acquisition (74.3 ± 15.6). Similarly, no significant difference was seen in BMI at three (20.9 ± 4.1), six (20.8 ± 3.6), and 12 months (22.1 ± 4.4) compared to baseline (20.2 ± 3.8) or the average of the year prior to *Bcc* acquisition (20.4 ± 3.1) (Fig. 2).

## Discussion

We describe the implementation of a *Bcc* eradication protocol for CF patients with a newly acquired *Bcc* colonization using an aggressive treatment regimen with a high success rate, suggesting its potential for benefit in this small group. Although no significant difference in lung function or BMI was observed at one year of follow-up between the eradication and control groups, the eradication group demonstrated universal *Bcc* clearance (100% as compared to 25% in the control group), stable lung function and nutritional status, and no treatment-related adverse events. We recognize the limitations of the small chronic Bcc case controls. This data is illustrative that chronically infected patients may have greater risk for exacerbation as well as increased CF-related morbidity. However, we acknowledge that these comparisons are not directly associated with the interventions outlined in our protocol. The protocol was relatively unbiased to patient selection, as all patients with newly discovered *Bcc* entered the protocol, with the exception of a single patient who was actively being treated for additional pulmonary pathogens, which precluded utilizing certain antibiotics.

It is important to note that a subset of patients, particularly younger patients, have been shown to spontaneously clear *Bcc* from sputum cultures suggesting that aggressive eradication may not always be necessary [13, 16]. Although it is not clear which patients are most likely to benefit from *Bcc* eradication, early eradication is likely to be clinically significant given the general association between *Bcc* colonization and progressive clinical decline [10, 17].

The protocol was, by intention, aggressive and it is not known whether this intensive therapy is required for successful *Bcc* eradication. Also note, this protocol demonstrated successful eradication of three different *Bcc* strains, some of which have not been previously associated with outcomes similar to an epidemic strain. Alternative protocols in pediatric patients with newly isolated *Bcc* have been described with some success and have utilized similarly aggressive regimens combining inhaled and intravenous Tobramycin, Ceftazidime and Temocillin [18]. The patients described by that series were not complicated by chronic *PsA* co-infection. An additional case-series described combination of inhaled amiloride and tobramycin with eradication of initial colonization in 3 of 4 patients but was unsuccessful in chronically infected patients [19, 20].

## Conclusion

This study has several limitations including a small sample size, retrospective single-center design, and a control group that consisted of chronically infected patients who might have a different course of disease progression. Therefore, a multi-center, prospective study is warranted to evaluate the efficacy of aggressive *Bcc* eradication and provide a more definitive evaluation for treatment of this severe complication of CF lung disease and associated potential benefits on CF-related morbidity and exacerbation frequency we observed compared to case controls.

## Abbreviations

*Bcc*: *Burkholderia cepacia complex*
BMI: Body mass index
CF: Cystic fibrosis
FEV_1_: Forced expiratory volume in 1 s
PsA: *Pseudomonas aeruginosa*
